# Prediction of complications in a high-risk cohort of patients undergoing corrective arthrodesis of late stage Charcot deformity based on the PEDIS score

**DOI:** 10.1186/s12891-015-0809-6

**Published:** 2015-11-14

**Authors:** Anica Eschler, Georg Gradl, Annekatrin Wussow, Thomas Mittlmeier

**Affiliations:** Department of Trauma, Hand and Reconstructive Surgery, University of Rostock, Medical Center, Schillingallee 35, D-18057 Rostock, Germany; Department of Trauma, Orthopedic and Reconstructive Surgery, Klinikum München Harlaching, Sanatoriumsplatz 2, D-81545 Munich, Germany

**Keywords:** Charcot feet, Diabetic neuroosteoarthropathy, DNOAP, Corrective arthrodesis, Surgical treatment, Complications, Diabetes mellitus, PEDIS classification

## Abstract

**Background:**

All diabetic neuroosteoarthropathy (Charcot arthropathy) treatment concepts are focused on a long-term infection-free, ulcer-free, and plantigrade sufficiently stable foot in order to avoid amputation. Reconstructive arthrodesis techniques for severe deformities are associated with high postoperative complication rates. This study reports a detailed complication analysis and provides a strategy that may help detect patients at risk for a complicated postoperative course.

**Methods:**

The study comprised 43 feet in 37 patients with severe non-plantigrade or unstable Charcot deformity, Eichenholtz stages II/III (Sanders and Frykberg types II-V), who underwent reconstructive arthrodesis of the mid- and/or hindfoot. Patients were retrospectively enrolled 4.5 years postoperatively (range 1.8–11.2 years). All patients showed at least two out of five positive Pinzur high-risk criteria (immuno-compromising illnesses, large bone deformity, longstanding ulcer overlying infected bone, regional osteopenia, obesity). Follow-up included a detailed clinical analysis and radiologic assessment with emphasis on complication analysis and evaluation in accordance to the PEDIS classification system.

**Results:**

Significantly lower overall complication rates, as well as re-operation, reulceration and amputation counts were found for patients with a cumulative PEDIS count below 7. For PEDIS single criteria, significantly lower overall complication rates were found for patients without signs of occlusive peripheral artery disease, an ulcer extent <0.9 cm^2^, ulcer depth including erosion and inflammation of the skin and subcutaneous tissues only. Soft-tissue complications affected 49 % of patients, hardware breakage 33 %, hardware loosening 19 %, non-union 18 % and amputation 21 %. Radiographs revealed a correct reconstruction and restoration of all foot axes postoperatively with partial recollapse at the lateral foot column; however, fixation strength for the medial column was maintained.

**Conclusions:**

Internal corrective arthrodesis for patients within the deformed stages of Charcot deformity can provide adequate reconstruction, as assessed by intraoperative radiographic measures, that exhibit superior long-term stability for the medial column. Despite a high risk patient population, a favourable outcome in terms of overall complication, re-ulceration, and amputation rates for patients/feet with a cumulative PEDIS count below 7 was found. The cut-off value of 7 may aid clinical decision-making during preoperative planning for Charcot deformity.

## Background

**Fig. 1:**
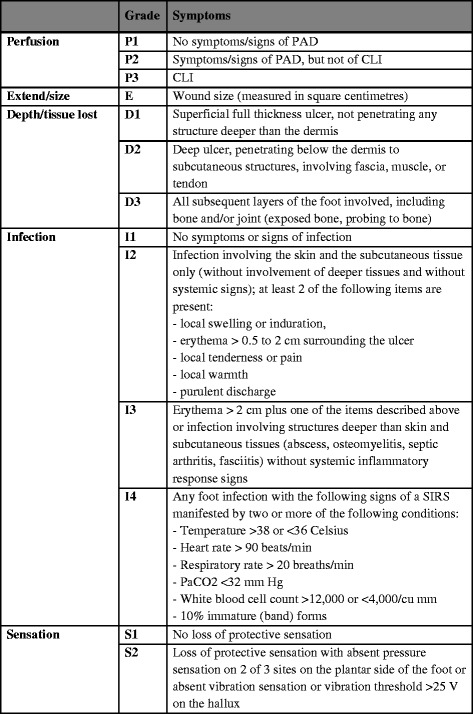
PEDIS classification system according to IWGDF Guidelines (International Working Group on the Diabetic Foot [5, 6]). Legend: PAD: peripheral arterial disease; CLI: critical limb ischemia; SIRS: systemic inflammatory response syndrome

The aim of any treatment concept involving neuroosteoarthropathy (Charcot arthropathy) for diabetic or other neuropathic diseases is a long-term infection-free, ulcer-free, and sufficiently plantigrade stable foot in order to maintain the ability to walk independently and avoid amputation [1, 2]. The search for the ideal treatment strategy is a challenge for foot and ankle specialists; furthermore, general evidence-based treatment algorithms are lacking and the literature is inconsistent regard to both the ideal treatment option and treatment timing because of the disease’s unrelenting progression. The disease progression may lead to a loss of osteo-ligamentous architecture and consequently loss of the plantigrade foot alignment; thus, inducing subsequent soft tissue complications such as skin breakdown, recurrent ulcerations, and infections [1, 3]. Neuroosteoarthropathic patients with chronic ulceration have a 12 times higher risk of amputation, compared to those with ulcer-free feet [4]. Ulceration characteristics are described by the widely-used PEDIS classification system developed by the International Working Group on Diabetic Foot (IWGDF) [5, 6]. It describes five specific ulcer criteria; perfusion, extent, depth/tissue loss, infection, and sensation; each criterion is graded by severity (Fig. 1).

With non-operative treatment with total contact casts or walkers, a long-term ulcer-free foot can be achieved in approximately 60 % of patients if plantigrade foot alignment can be established (early stage I/II applying the Eichenholtz classification system) [1]. Patients with an advanced degree of deformity and mid- and/or hind-foot instability experience less favourable results after non-operative treatment. Multiple recastings and prolonged disability are the consequences, and 40–51 % of Charcot patients may need surgical treatment at some point in the disease’s progression [1, 7, 8]. At this time point, when signs of instability and progressive malalignment are present, reconstruction arthrodesis techniques are indicated [1, 9, 10]. Reconstruction arthrodesis techniques vary from external fixation techniques such as ring fixators to internal fixation techniques such as plates, screws, or bolts (or combinations of these techniques) [1, 2, 9, 11, 12]. Due to the absence of general algorithms, the treatment option is usually determined by individual patient factors and the surgeon’s expertise [1].

For surgery in Charcot disease patients, prolonged healing periods, high rates of infectious complications, non-union and malunion, stress fractures, fixation failure, metal-induced soft-tissue irritation, implant breakage or loosening, and concomitant high re-operation rates are frequently described [1, 7, 11–18]. Due to these multiple disadvantages, in 2007 Pinzur et al. [1, 15] proposed that patients with high risk criteria such as a large bone deformity, an longstanding ulcer overlying infected bone, regional osteopenia, obesity, or immunocompromising illnesses do not qualify for open reduction and internal fixation. Instead, in these cases, percutaneous correction and fixation with an external ring fixator is recommended. Only in cases of available low-risk criteria (no open wounds, no history of deep infection, good bone quality, minimal diabetes-associated comorbidity, no morbid obesity), internal corrective arthrodesis is recommended [1, 15]. However this recommendation was based on the experience of one single surgeon and no detailed algorithm was provided [1]. Thus, it remains unclear how many high- or low-risk criteria are applicable to achieve the desired outcome.

In view of the foregoing, we retrospectively evaluated a consecutive series of neuroosteoarthropathic patients who underwent corrective arthrodesis of late stage Charcot mid- and hind-foot neuroosteoarthropathy with at least two out of the five positive Pinzur high-risk criteria. A 4.5 years follow-up was conducted to determine whether internal corrective arthrodesis could provide the desired outcome of a foot that was infection-free and ulcer-free for a long period, despite the presence of positive high risk criteria. The results were then quantified based on the PEDIS criteria in order to evaluate those patients that may qualify for internal corrective arthrodesis despite Pinzur high-risk criteria.

## Methods

The study comprised 43 feet in 37 patients with severe Charcot neuroosteoarthropathy who underwent internal corrective arthrodesis from November 2005 to March 2012 and were retrospectively reviewed. The indication criteria for corrective arthrodesis were: (1) a clinically and radiographic non-plantigrade foot alignment; (2) a high degree of instability of the medial or/and lateral midfoot and/or hindfoot region proven by clinical examination and visualized by the changes of radiologic axes as described by Sammarco et al. [19]: positive talar-first metatarsal angle in anterior-posterior (AP) views, negative talar-first metatarsal angle in lateral views, negative calcaneal-fifth metatarsal angle in lateral views, and deviations from the neutral axis in Saltzman views; (3) the foregoing, plus clinically manifest or impending ulceration of soft tissues overlying bony deformity; and (4) failed primary conservative treatment.

The mean patient age (24 males; 13 females) was 56.7 ± 8.5 years (range 29–76). All but two patients suffered from type 2 diabetes (*n* = 31) or type 1 diabetes (*n* = 4) with insulin dependency in 94 % of the cases (*n* = 33). Twenty-eight patients (76 %) suffered from three or more than three secondary diagnoses. Thirty-three feet (77 %) were operated during the consolidation phase (Eichenholtz stage III). Ten feet (23 %) presented with stage II arthropathy (coalescence). Applying the topographic classification, according to Sanders and Frykberg, a mid-foot affection type II and/or III, corresponding to the Lisfranc and Chopart joint region, was visible in all feet; however, 15 radiographs showed an additional involvement of the subtalar and talocalcaneal joints, according to Sanders and Frykberg type IV.

The preoperative assessment included anterior-posterior and lateral weight-bearing radiographs of the foot and ankle joint, including Saltzman views based on the following described angles. In case of doubt regarding the peripheral vascular status, color-coded duplex sonogram was performed before surgery to reveal patients with relevant macroangiopathy. Subsequently, angiographic dilatation and stenting was performed in two patients preoperatively.

The location of the corrective osteotomy and arthrodesis was determined according to radiologic and clinical assessment, and stabilization was performed using specific implants in order to achieve maximum stability. The procedure was as follows. A medial utility incision exposed the medial column as well as the talonavicular, naviculocuneiform, and tarsometatarsal joints for either osteotomy, excision of the bony deformity, or denuding the joint surfaces from cartilage was performed as well as resection of necrotic bone; this was done in preparation for fusion and reconstruction of a plantigrade foot position. Stabilization of the medial column was performed in 21 feet (49 %) using extramedullary devices, of which 9 feet (21 %) received angular stable plates and 9 feet (21 %) received intramedullary implants only. Seven feet (16 %) were stabilized using a combination of intra- and extra-medullary implants (Table 1). With isolated collapse of the medial column, the hindfoot and lateral foot region remained untouched. Additional stabilization of the lateral column was performed in 22 feet (51 %); 6 of these feet (14 %) were stabilized with angular stable plates. Hindfoot arthrodesis was performed in 16 cases (37 %) using compression screws (*n* = 9; 21 %), angular stable plates (*n* = 6; 14 %) or locking nails (*n* = 1; 2 %). In order to reconstruct the osseous foot geometry, resection of the necrotic midfoot/hindfoot bones was necessary in 7 feet (16 %). In 30 cases (70 %), osseous defects were filled with autologous iliac crest grafts. One case (2 %) required lengthening of the gastrocnemius-soleus complex. In 3 cases (7 %), Achilles tendon lengthening via the Dockery technique was performed as indicated by intraoperative evaluation of tightness of the Achilles and gastrocnemius tendon complex.

**Table 1:** Surgical procedures performed on study group patients

Surgical approach/Point of stabilization		All	
		N	%
Medial column	All	37	86
	Extramedullary	21	49
	Intramedullary	9	21
	Combined	7	16
Lateral column	All	22	51
	Standalone	-	-
	Combined to medial column/hindfoot stabilization	22	51
Hindfoot	All	16	37
	Standalone	6	14
	Combined to medial/lateral column stabilization	11	26
Additive osseous resection		7	16
Additive autologous iliac crest graft		30	70

Postoperatively, a lower leg splint was applied and replaced by a total contact cast with soft tissue consolidation. Mobilization was performed with partial weight-bearing of 20 kg if possible. Routine follow-up e.g., for cast replacement and radiographs, was conducted two weeks after hospital discharge and then at monthly intervals until radiographic bony consolidation was proven. Subsequently annual follow-up visits were established. Follow-up ended if amputation occurred. Mean follow-up averaged 4.5 ± 6.6 years (range 1.8–11.2).

The 4.5 years follow-up included a detailed failure analysis for the peri- and post-operative time period, focusing on complication and re-operation rates. Therefore, early (30 days postoperatively), intermediate (30 days to 5 months postoperatively) and late complications (from postoperative month 6 onward) were recorded and assessed according to their degree of severity and the need for either further surgery or conservative regime. All patients were then graded according to the PEDIS classification system [5, 6, 20] and Pinzur criteria [1, 15]. The former, developed by the International Working Group on the Diabetic Foot (IWGDF), describes the diabetic foot depending on severity of the following characteristics: lower leg/foot perfusion, ulcer extent, ulcer depth/tissue loss, infection, and sensation [5, 6, 20]. The latter, published by Pinzur in 2007 [1, 15], distinguishes high-risk criteria as a large bone deformity, a longstanding ulcer overlying infected bone, regional osteopenia, obesity, or immuno-compromising side illnesses, as well as low-risk criteria such as absence of open wounds, no history of deep infection, good bone quality, minimal diabetes-associated comorbidity, and absence of morbid obesity. On the basis of this Pinzur grouping [1, 15] and PEDIS criteria [5, 6, 20] a complication analysis was performed with the goal of evaluating those patients at risk who do not qualify for internal corrective arthrodesis. Furthermore, patient mobilization and casting length were registered.

A detailed radiologic follow-up included measurement of the AP talar-first metatarsal angle, lateral talar-first metatarsal angle, calcaneal-fifth metatarsal angle, dorsal midfoot displacement in pre-, post- and follow-up radiographs. The mean values are given in absolute numbers representing the change of angulation for the AP talar-first metatarsal angle; however, the range is given with algebraic signs; thus, negative values correspond to abduction deformity and positive values correspond to adduction deformity of the forefoot. Twenty-nine feet were available with complete radiologic follow-up.

### Ethics, consent and permissions

The study was approved by the local ethical committee under the document no. A 2014–0174 (2014/11/27) and is in accordance with the declaration of Helsinki. All patient’s gave consent to participate.

### Statistical analysis

Results are given as mean ± SEM (range). After satisfying the assumption of normality (Kolmogorov), the paired *t*-test analysis or the Mann–Whitney *U* Test (non-normal distribution) were performed to analyse the differences in radiographic parameters. For differences in complication rates, according to preoperative PEDIS single criteria (“P” perfusion, “E” extent, “D” depth, “I” infection, “S” sensation); cumulative PEDIS count groups were developed and a paired *t*-test analysis was performed. Complication counts, re-operation and re-ulceration rates, amputation counts, and length of immobilization were analysed in relation to the cumulative PEDIS count by comparing the group below the boundary value as defined above and hereafter. The boundary value was initially set as the cumulative PEDIS mean value (6.5 ± 2.3), then, one SEM value (8.8) was added to the mean value, and then two SEM values (11.1) were added to the mean value. Significance was defined at *p* < 0.05. For assessment of complications, re-operation, re-ulceration, number of amputations, and length of immobilization dependent upon lower leg/foot perfusion, P1 was compared to P2 and P3. Dependent upon the depth of ulceration, D1 was compared to D2 and D3 (and D1 and D2 were compared to D3). Dependent upon the presence of local infection, I1 was compared to I2, I3, and I4 (and I1 and I2 were compared to I3 and I4). Sensation was not further considered since all patients showed a loss of protective sensation (S2). For the ulcer extent, results were compared based on the cumulative PEDIS value (ulcer extent as mean 0.9 ± 0.2 cm^2^). Statistical analysis was performed using IBM SPSS Statistics version 20.0 software (Armonk, New York, USA).

## Results

### Complication analyses

**Fig. 2:**
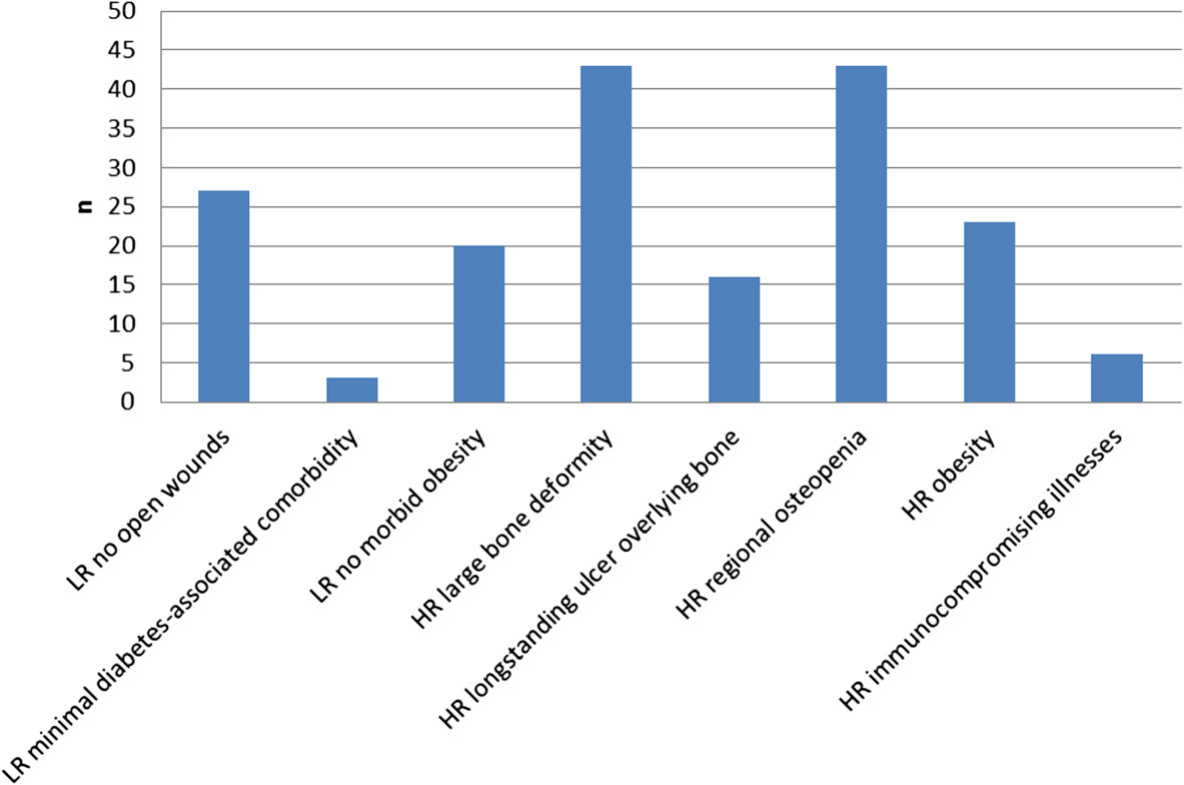
Classification according to Pinzur’s criteria [1, 15]

All patients/feet showed a minimum of 2 out of 5 positive Pinzur high-risk criteria (mean 3.1 ± 0.1; range 2–5, Fig. 2). According to Pinzur [15], all feet were at high risk for postoperative complications. The following complication analyses focused on soft tissue complications, implant associated complications, non-union (stable/non-stable) and amputation; these complications were determined during the first run of the analyses. Almost half (20 of 43 feet; 46.5 %) developed a mean 0.51 ± 0.1 early complications, 26 feet (61.0 %) developed intermediate complications (mean 0.81 ± 0.1), and 31 feet (72.1 %) developed late complications (mean 1.79 ± 0.2).

Soft tissue complications occurred in 34 feet (79 %) of 29 patients (67 %) during the follow-up period. Specifically, 21 feet (49 %) developed superficial wound infections, 16 feet (37 %) developed (re)ulceration, and 15 feet (35 %) had impaired wound healing. Osteomyelitis affected 4 feet (9 %). About half (52 %; *n* = 40) of the soft tissue complications occurred during the late follow-up phase, 27 % (*n* = 21) developed within the early phase, and 21 % (*n* = 16) occurred within the intermediate phase.

Hardware-associated complications affected 22 feet (51 %) of 21 patients (49 %); hardware breakage, which might affect single screws, occurred in 14 feet (33 %) and hardware loosening occurred in 8 feet (19 %). In 8 feet (18 %), non-union of the arthrodesis region was observed. Both hardware-associated complications and non-union occurred most frequently in the late phase (54 %); however, 42 % were observed within the intermediate phase.

Amputation had to be performed on 9 patients (21 %), with a need for lower leg amputation in 6 cases (14 %), forefoot Chopart amputation in one case (2 %), and toe amputation in 2 cases (5 %). Amputation was performed at a mean 1.1 ± 0.8 years (range 0–3) after initial surgery.

For complication management, 1.9 ± 4.0 (range 0–5; 64 %) complications of a mean 2.9 ± 3.5 complications (range 0–7, *n* = 131) had to be resolved surgically. In 14 cases (33 %), only minor revision surgery such as soft-tissue debridement or jet lavage were necessary. In 20 cases (47 %), major surgery as rearthrodesis or amputation was performed, among which 16 cases (37 %) underwent minor revision surgery during follow-up as well (Table 2). This resulted in a mean of 3.1 ± 5.0 (range 0–10) revision surgeries. Regarding the time point of complication resolution, 18 % of early complications, 25 % of intermediate complications, and 57 % of late complications required surgical complication management.

**Table 2:** Type of revision surgery performed on study patients

Revision surgery	All		Early		Intermediate		Late	
	n	%	n	%	n	%	n	%
Corrective Arthrodesis	24	55.8	1	2.3	7	16.3	16	37.2
Local osseous resection	2	4.7	-	-	1	2.3	1	2.3
Implant removal	18	41.9	-	-	10	23.3	8	18.6
Amputation	9	20.9	1	2.3	2	4.7	6	13.9
Others^a^	34	39.1	14	16.1	4	4.6	16	18.4

^a^soft tissue debridement, jet lavage, antibiotic chain placement/replacement, haematoma evisceration, split-skin grafting, closed amputation, muscle or musculocutaneous flaps, fracture osteosynthesis

### Complication analyses considering PEDIS criteria

Taking into consideration the preoperative cumulative PEDIS count and the single PEDIS criteria (Fig. 1), a differentiated analysis was performed to ascertain those high-risk patients who qualify for internal corrective fixation techniques, despite a Pinzur high risk rating.

**Fig. 3:**
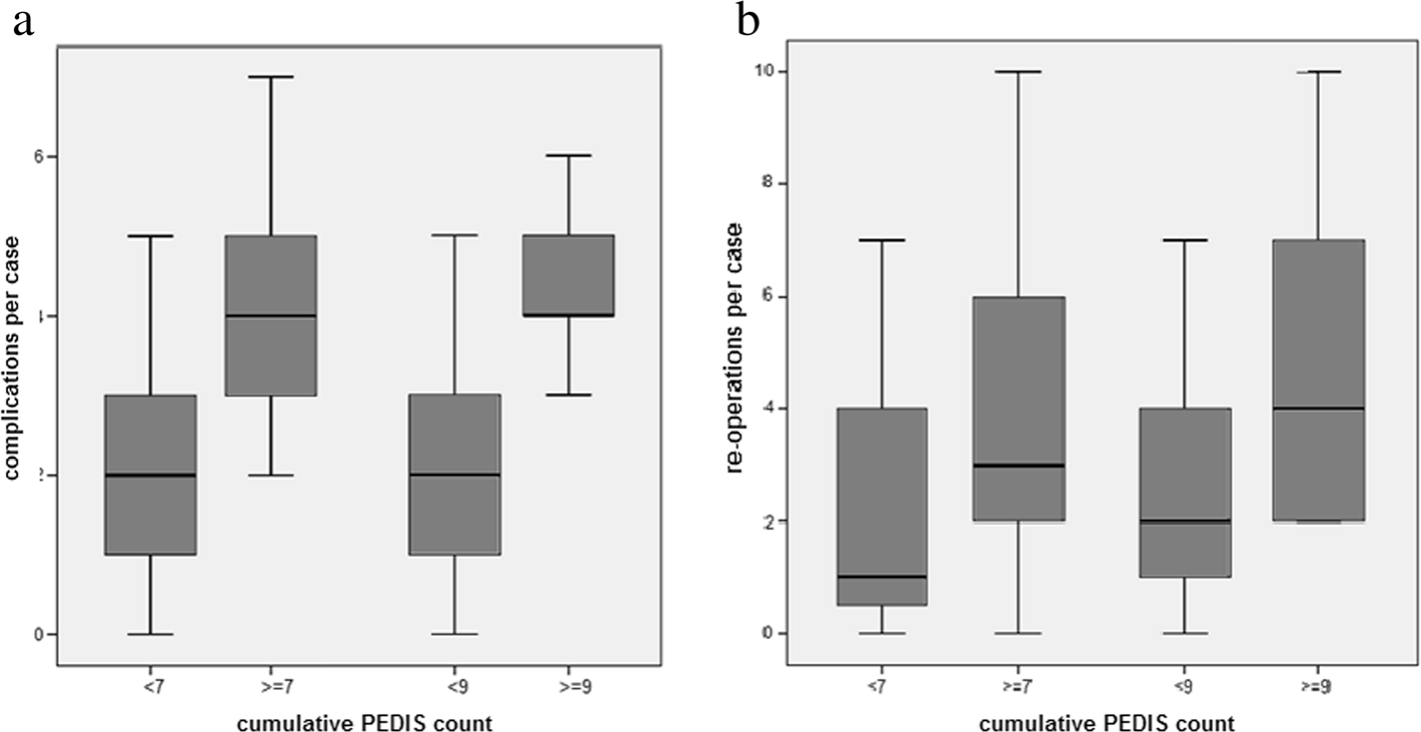
Overall complication rates **(a)** and re-operation rates **(b)** according to the cumulative PEDIS count [5, 6; Fig. 1]

The mean cumulative PEDIS count accounted for 6.5 ± 2.3 (range 0–14). When comparing the groups with ≥7 and <7 cumulative PEDIS counts, a significantly lower overall complication rate for the <7 group (*p* < 0.01), as well as a significant lower re-operation (*p* < 0.05), re-ulceration (*p* < 0.03) and amputation counts (*p* < 0.01) were revealed (Fig. 3a). By differentiating early, intermediate, and late complications only the latter (late) were significantly reduced (*p* < 0.01). When adding one SEM to the mean value (6.5 + 2.2 = 8.8); thus, comparing the groups ≥9 and <9, cumulative PEDIS value analogue results were achieved as shown in Fig. 3b. Only the criterion of re-ulceration showed non-significant results. When adding two SEM to the mean value (8.8 + 2.2 = 11.0); thus, comparing the groups ≥11 and <11 cumulative PEDIS values, no significant differences were found. This reveals a higher risk for complications among patients with a cumulative PEDIS count ≥7.

**Fig. 4:**
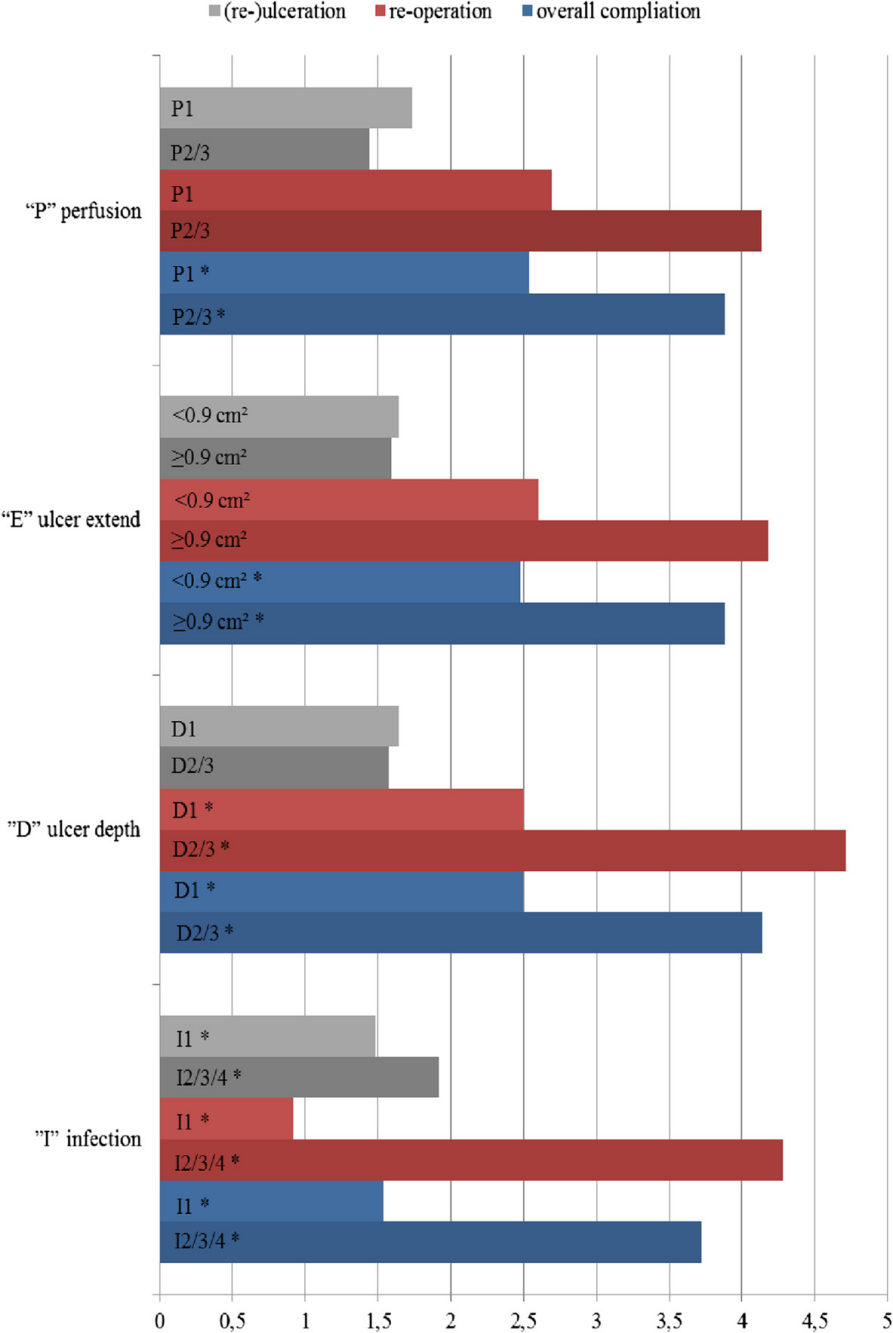
Complication evaluation applying the PEDIS classification system [5, 6]. Legend: *significant values (*p* < 0.05)

The analyses of PEDIS under the single criterion of perfusion (“P”) showed a significantly lower overall complication rate when no symptoms or signs of peripheral artery occlusive disease (PAD) were apparent when compared to feet with symptoms or signs of PAD or manifest critical limb ischemia (CLI) (P1 vs. P2 and P3; *p* < 0.05; Fig. 4). In addition, apparent complications were significantly more amenable to successful conservative treatment (*p* < 0.05).

For the ulcer extent (“E”; 8.8 ± 0.2 cm^2^; range 0–6), significantly lower overall complication rates (*p* < 0.01) and amputation rates (*p* < 0.05) were found for a preoperative ulcer extent of <0.9 cm^2^. Again, apparent complications were significantly more amenable to successful conservative treatment (*p* < 0.05).

Applying the criterion of ulcer depth/tissue loss (“D”) significantly lower overall complication rates (*p* < 0.01), re-operation rates (*p* < 0.03) and amputation rate (*p* < 0.01) were found with the comparison of superficial full-thickness ulcers/erosions (grade 1) to ulcers penetrating below the dermis (grade 2) or ulcers penetrating all deeper layers of the foot, including bone/joints (grade 3) (D1 was compared to D2 and D3). Again, apparent complications were significantly more amenable to successful conservative treatment (*p* < 0.01).

The criterion of infection (“I”) revealed significant results in all parameters when comparing feet with no signs of infection (I1) to any signs of infection (I2, I3 and I4), complication rate (*p* < 0.01), re-operation rate (*p* < 0.01), re-ulceration rate (*p* < 0.01), and amputation rate (*P* < 0.05). Comparing those with inflammation of the skin and subcutaneous tissue (I1 and I2) to those with extensive erythema deeper than the skin and subcutaneous tissue (I3), only the overall complication rate reached persistent significant differences (*p* < 0.01).

### Radiological results

The mean talar-first metatarsal angle in an AP view of the foot, as an indicator for abduction/adduction midfoot deformity, improved from 8.4 ± 4.2° (range –17 to +31) preoperatively to 4.6 ± 3.3° (range –8 to +23) postoperatively, but slightly decreased to 5.6 ± 3.9° (range 24 to +17) at final follow-up. An effective 3.8 ± 5.5° (range –11 to +22) correction to the neutral (0°) axis was achieved postoperatively; it decreased slightly to 2.8 ± 6.5° (range –18 to +41) at final follow-up. Full correction to the anatomic neutral position (0°) axis was observed in 83 % (*n* = 24) of the feet. In 38 % of patients (*n* = 11) an overcorrection with change of algebraic sign was observed. Due to the observed recollapse, 66 % (*n* = 19) of patients showed correct AP talar-first metatarsal angle at final follow-up (Table 3).

**Table 3:** Radiographic evaluation

Radiographic angle		mean	min	Max	SEM	p*
talar-first metatarsal angle (AP)^a^	pre-surgery	8.4	−17.1	30.8	4.2	-
	post-surgery	4.6	−7.9	23.0	3.3	
	final follow-up	5.6	−23.5	17.0	3.9	
	Correction post-surgery	3.8	−10.7	21.8	5.5	
	Correction final follow-up	2.8	−17.5	40.6	6.5	
talar-first metatarsal angle (lat.)^a^	pre-surgery	−12.5	−28.5	14.9	8.8	*a <0.01, *b <0.01, *d <0.01
	post-surgery	2.5	−11.8	28.1	7.3	
	final follow-up	−5.9	−28.2	18.5	8.8	
	Correction post-surgery	15.0	−9.2	33.8	7.6	
	Correction final follow-up	6.6	−33.2	17.5	7.7	
calcaneal-fifth metatarsal angle lat.)^a^	pre-surgery	10.8	−8.1	29.0	7.5	*a <0.01, *b <0.01, *d <0.01
	post-surgery	18.0	0.1	40.7	6.9	
	final follow-up	9.0	−11.8	33.0	5.2	
	Correction post-surgery	7.2	−7.9	21.5	7.1	
	Correction final follow-up	−1.8	−15.5	15.6	8.9	
dorsal midfoot displacement^b^	pre-surgery	16.7	0.0	34.7	7.0	*a <0.01, *c <0.05
	post-surgery	7.2	0.0	28.7	4.3	
	final follow-up	10.8	1.6	33.6	5.2	
	Correction post-surgery	9.5	−8.8	32.4	9.2	
	Correction final follow-up	5.9	−21.6	25.8	9.4	

*significant values; *a pre-surgery vs. post-surgery’ *b post-surgery vs. follow-up; *c pre-surgery vs. follow-up; *d correction post-surgery vs. correction final follow-up

^a^ Values given in °

^b^ Values given in mm

The mean lateral talar-first metatarsal angle showed significant improvements of +15.0 ± 7.6° (range: –9 to +34; *p* < 0.01) postoperatively, compared to preoperative values. At final follow-up an improvement of +6.6 ± 7.7° (range –33 to +18; *p* < 0.01) was found; this loss of reduction was significant. Ideally, the intraoperative change of the lateral talar-first metatarsal angle exhibited a high positive value, corresponding to a change from a valgus deformity to a neutral axis, therefore, erection of the foot arch. This was achieved in 96.6 % (*n* = 28) of the patients postoperatively and was still observed in 62.1 % (*n* = 18) at final follow-up.

The calcaneal-fifth metatarsal angle in the lateral view displaying the lateral foot arch is optimally erected to intraoperative positive values. The calcaneal-fifth metatarsal angle improved significantly with +7.2 ± 7.1° (range –8 to +22; *p* < 0.01) when the pre- and post-operative values in our study population were compared; however, it decreased to –1.8 ± 8.9 (range –16 to +16; *p* < 0.01) at final follow-up, thus, denoting a recollapse of the lateral foot arch in mean. However, postoperatively, 93 % (*n* = 26) of patients showed correct reduction of the lateral foot arch, and ultimately, 35 % (*n* = 10) showed correct reduction at final follow-up.

Since standard angles on lateral radiographs tend to underestimate the grade of deformity with midfoot joint displacement [19], the dorsal midfoot displacement (lateral view x-ray; the vertical distance at the level of dislocation between the talar midline axis and the first metatarsal axis) was measured. Dorsal midfoot displacement showed significant postoperative improvements of +9.5 ± 9.2 mm (range –9 to +32; *p* < 0.01) and slight bone loss; however, a +5.9 ± 9.4 mm correction (range 22 to +26; *p* < 0.05) was observed at final follow-up. This means that in 83 % (*n* = 24) of the patients postoperatively, and ultimately 59 % (*n* = 17) at final follow-up achieved adequate correction.

### Hospitalization and mobilization

Mean hospitalization duration following the initial surgery was 28.3 ± 14.4 days; the minimum duration of stay was 8 days, the maximum 87 days caused by a very complicated course of one patient. The mean postoperative casting period was 2.5 ± 3.7 months (range 0.3–7 months).

At final follow-up, 60 %, of the patients (*n* = 26) were fully mobile without a need for walking aids; 7 patients (16 %) used non-orthopaedic shoes and 19 patients (44 %) used orthopaedic shoes. Therefore, the goal of long-term walking independence was reached. Seven patients (16 %) wore a prosthesis. Only 1 patient (2 %) used a wheelchair, 2 patients (5 %) used a walker, and 5 patients (12 %) used a cane.

## Discussion

For diabetic neuroosteoarthropathy treatment general evidence-based treatment algorithms are lacking and the literature is inconsistent regarding both the ideal treatment type and timing of treatment due to its unrelenting progression. With disease progression and unstable non-plantigrade alignment of the mid- and hind-foot, a high degree of skin breakdown and ulceration at the site of bony deformities occurs [1, 3]. Pinzur [1, 8] and other authors [21, 22] proposed the main goals: an infection-free and ulcer-free foot for a long duration together with the ability to use commercially-available depth-inlay shoes and custom-accommodative foot orthoses for maintaining long-term walking independence. Non-surgical measures such as total contact casting represent the treatment option of choice in cases of plantigrade foot positioning [19, 23, 24]. However, the progressive character of instability is associated with a serious impairment of the quality of life and an estimated risk of up to 49 % to develop recurrent ulceration together with a high risk for further complications such as infections or eventually amputation [23, 25]. Therefore, early reconstructive surgery is suggested by several authors as a valuable treatment option in the presence of severe deformity [25–28]. Evidence-based literature does not exist, but clinical reports stress that stability can be restored with precise surgical technique, appropriate perioperative education, and postoperative therapy (assuming adequate patient compliance) [2, 27]. Nevertheless, according to Pinzur [1], certain high-risk criteria such as a large bone deformity, a longstanding ulcer overlying infected bone, regional osteopenia, obesity, or immuno-compromising illness showed increased complication rates and therefore interfere with open reduction and internal fixation. Instead, percutaneous correction and fixation with an external ring fixator is recommended for high-risk constellations

In patients with low-risk criteria, internal corrective arthrodesis is recommended [1]. Low-risk criteria include absence of open wounds, no history of deep infection, good bone quality, minimal diabetes-associated comorbidity, and absence of morbid obesity. Actually, the diagnosis of Charcot deformity is frequently delayed and only made in the deformed stages [29]. Conversely, despite prompt immobilization and protected weight-bearing, some patients develop severe deformities [29].

We retrospectively reviewed 43 feet with a deformation stage consistent with Charcot disease and determined as a high-risk group, which had at least two positive high-risk criteria according to Pinzur [1]. During a 4.5 years follow-up, a detailed complication analysis was performed which revealed high complication rates. The early postoperative period was characterized by soft tissue complications in which 75 % of patients were affected by immediate postoperative wound infections (47 %); this finding may be attributable to the endangered diabetic patient population [3] and concurs with rates of 7–26 % reported by other studies with an mean of 2.5 years of follow-up [13, 23]. The osteomyelitis rate was 9 % in our study population. It is reported to range from 12 % to 16 % after reconstructive surgery in other studies [13, 30]. However, our osteomyelitis rate is markedly lower than the 33 % osteomyelitis rate after conservatively-treated diabetic foot infections [31].

About one third (31 %) of the patients suffered from hardware breakage and 18 % developed hardware loosening with consecutive non-union. Sammarco et al. [19] observed similar rates with 32 % implant breakage in their 4.3 years of follow-up after midtarsal arthrodesis. A recent review [2], including 95 Level IV and V studies, revealed similar results and ulcer-free feet in most cases; however, it described a 22.4 % non-union rate. Implant or technique-associated problems, as well as the prolonged healing period in the altered diabetic metabolism, are likely to be causal [32]. Nevertheless, currently there is consensus that even with incomplete union, an ulcer-free plantigrade foot may be achieved [2]. We also observed 9 cases (20 %) with amputation, resulting in an annual amputation rate of 4.4 %, which concurs with Saltzman et al. [25] who reported on a cohort of 115 patients with a mean of 3.8 years of follow-up; they found an annual amputation risk of 2.7 % of cases without ulceration and an amputation risk of 28 % in cases with ulceration. These cases incurred a 23 % risk of requiring more than 18 months of casting and a high risk of about 49 % for recurrent ulceration. In our study population, 36 % of the patients suffered from recurrent ulcerations. Illgner et al. [11] reported on 205 patients with an external fixator and a follow-up period of 21 months; they found a 25 % re-ulceration rate. A study of 115 conservatively treated patients with 3.8 years of follow-up by Saltzman et al. [25] showed 49% re-ulceration rates. Altogether, we observed 2.9 complications/foot leading to the question: “Can those patients at risk for a complicated postoperative course be detected in preoperative planning?”.

In view of the foregoing we developed a potential predictor that would reveal those high-risk patients qualifying for internal corrective arthrodesis despite this risk. It was to take the PEDIS single group of criteria into account: feet without signs of PAD, an ulcer extent <0.9 cm^2^, an ulcer depth up to erosion, and inflammation including only skin and subcutaneous tissue. This group of criteria alone showed significantly lower overall complication rates (P1, E1, D1, I2, (S)). For this degree of ulceration, the data also significantly predict lower amputation rates and for this degree of ulceration depth, they significantly predict lower amputation and reoperation rates. Since all factors but sensation showed significant influences on complication rates, we think that the cumulative PEDIS count rather than the PEDIS single criteria is practicable for clinical use because the former takes the entire patient into consideration. This study significantly revealed a favourable outcome in terms of overall complication, re-ulceration, and amputation rates for patients/feet with a cumulative PEDIS count below 7. The cutoff value of 7 may aid clinical decision-making in preoperative planning for a Charcot deformity and an internal corrective arthrodesis. By implication, these results endorse the importance of early diagnosis for optimal treatment planning.

In regard to radiologic evaluation, the goal of reconstruction is to correct the non-plantigrade foot position and to establish a neutral AP tarso-first metatarsal angle, a positive lateral tarso-first metatarsal angle, a calcanear-fifth-metatarsal angle, and a dorsal midfoot displacement correction to normal values. Correction was completely achieved in 83 % of the AP talar-first metatarsal angle, in 97 % of patients for the lateral talar-first metatarsal angle, 90 % of patients for the calcaneal-fifth metatarsal angle and 83 % of patients with dorsal midfoot displacement; these findings concur with those of other studies [19, 30]. In addition, at final follow-up a significant recollapse of the longitudinal foot arch for both the medial column and, to a greater extent, the lateral column had to be observed in order to minimize the number of patients with persistent correction to 62 % medially and 35 % laterally. Correction of AP talar-first metatarsal angle recollapsed in 17 % of feet. Recollapse of the midfoot, measured by the dorsal midfoot displacement, lowered radiographic success rates to 59 % of the patients. Wiewiorski et al. [30] similarly experienced the worst recollapse rate for the lateral tarso-first metatarsal and calcanear-fifth-metatarsal angles. Those radiographic measurements reveal a good overall reliability for reconstructive techniques in our patient population. The significance of those radiographic measurements in regard to patient satisfaction is unknown [30]; this adds to the difficulty of radiographic evaluation, due to its putative measurement error, to determine weaknesses of this study. In addition, we noted another limitation: patient satisfaction was not evaluated in this study. This limitation is of significant importance. By forming groups based on clinically defined boundaries, heterogeneous cohorts may result, thus, leading to a statistical error. The study design was retrospective with specific inclusion criteria. Therefore, the limited patient sample negatively impacted generalizations.

## Conclusions

Internal corrective arthrodesis in the more advanced stages of Charcot deformity can provide adequate reconstruction with intraoperative radiographic measurements. Superior stability was achieved for the medial column in this 4.5 years follow-up period with the lateral column showing less stability and a tendency to recollapse. Despite the favourable radiologic results, overall complication rates were high. A good predictor implementing Pinzur and PEDIS criteria was developed to identify those high-risk patients qualifying for uncomplicated internal corrective arthrodesis. Despite two-to-five positive Pinzur high-risk criteria, a favourable outcome in terms of overall complication, re-ulceration, and amputation rates for patients/feet with a cumulative PEDIS count below 7 was found. The cutoff value of 7 may aid clinical decision-making during preoperative planning for Charcot deformity and internal corrective arthrodesis.
